# Conventional and Advanced Processing Techniques and Their Effect on the Nutritional Quality and Antinutritional Factors of Pearl Millet Grains: The Impact on Metabolic Health

**DOI:** 10.3390/antiox14121460

**Published:** 2025-12-05

**Authors:** Letícia da Silva Oliveira Moura, Rita de Cássia Avellaneda Guimarães, Aline Carla Inada, Juliana Rodrigues Donadon, Arnildo Pott, Rosângela dos Santos Ferreira, Carolina Di Pietro Fernandes, Caroline de Moura Costa, Fernando dos Santos Moura, Karine de Cássia Freitas, Danielle Bogo, Valter Aragão do Nascimento, Priscila Aiko Hiane

**Affiliations:** 1 Graduate Program in Health and Development in the Central-West Region of Brazil, Medical School, Federal University of Mato Grosso do Sul, Campo Grande 79070-900, MS, Brazil; silva.leticia@ufms.br (L.d.S.O.M.); inada.aline@gmail.com (A.C.I.); rosangela.ferreira@ufms.br (R.d.S.F.); nutricaroldipietro@gmail.com (C.D.P.F.); karine.freitas@ufms.br (K.d.C.F.); danielle.bogo@ufms.br (D.B.); valter.aragao@ufms.br (V.A.d.N.); priscila.hiane@ufms.br (P.A.H.); 2 Pharmaceutical Science, Food and Nutrition Faculty, Federal University of Mato Grosso do Sul, Campo Grande 79070-900, MS, Brazil; juliana.donadon@ufms.br (J.R.D.); arnildo.pott@gmail.com (A.P.); caroline.moura@ufms.br (C.d.M.C.); 3 Graduate Program in Quality Management Technology, Unigran, Campo Grande 79010-190, MS, Brazil

**Keywords:** pearl millet, food processing, nutritional quality, antinutritional factors, metabolism

## Abstract

Food processing techniques are widely used in the food industry to ensure food safety, extend shelf life, and enhance sensory appeal without compromising the product’s nutritional quality. Pearl millet, which is considered a “nutricereal”, features essential content of proteins, soluble and insoluble fibers, minerals (e.g., iron, zinc, and magnesium), bioactive compounds (e.g., phenolic acids, flavonoids, and carotenoids), and antinutritional factors (e.g., phytic acid, C-glycosyl flavones, tannins, and non-digestible oligosaccharides). This nutricereal also undergoes processing methods to improve or maintain its nutritional quality while simultaneously reducing antinutritional factors. Pearl millet processing techniques are categorized into conventional (or traditional) and advanced methods; however, a knowledge gap exists in studies evaluating the post-processing of pearl millet and its impact on metabolic health in in vivo and in vitro experimental models. This study aims to demonstrate the principal conventional and advanced processing techniques used in pearl millet, how they can ensure nutritional quality and reduce antinutritional factors, and how the final post-processing product could impact metabolic health.

## 1. Introduction

The main goal of nutrition is to prevent and treat nutritional deficiencies. However, when nutrition is excessive, the body must contend with problems related to the quantitative control of nutrient absorption and storage. Consequently, the excessive absorption and accumulation of energy substances, particularly carbohydrates and lipids, can lead to metabolic diseases, such as obesity, diabetes, hyperlipidemia, and certain types of cancer [1]. Another important group is micronutrients, such as vitamins and minerals, which are essential to metabolic health, tissue function, and immune system support [2].

The growing consensus among health-conscious individuals is driving them to seek out foods with greater nutritional value, such as whole foods and healthy options, prompting food manufacturers to focus on developing functional foods [3]. Thus, the food industry began to gain greater recognition for functional bioactive compounds in various processed products while also maintaining their functional and phytochemical properties in human foods [3].

Functional foods are conceptualized as those that contain one or more functional components, such as polyphenols, antioxidants, carotenoids, prebiotics, probiotics, sterols and/or other components with some functional properties and that provide essential functions in the body, promoting health or preventing the development of chronic non-communicable diseases (NCDs), such as cardiovascular diseases, diabetes mellitus and cancer [3].

Pearl millet (*Cenchrus americanus* (L.) Morrone) or *Pennisetum glaucum* (L.) Br. is known as “nutricereal” and is a significant subsistence crop, particularly in arid and semi-arid regions of Africa and Asia [4]. This crop is characterized by tolerance to saline stress, making it a suitable forage option in the semi-arid areas [4]. In Brazil, pearl millet has been cultivated for approximately 50 years; however, it is mainly consumed in animal nutrition. In 2022, ANVISA (Agência Nacional de Vigilância Sanitária), the Brazilian health surveillance agency, approved the human consumption of pearl millet as a whole food. Consequently, scientific research has explored it as a potential functional food for human nutrition in Brazil [5,6].

This cereal has been recognized as a functional food in Asian and African countries due to its high nutritional value, being a rich source of nutrients [7]. The pearl millet grains are rich in carbohydrates (65–75%) [8,9], soluble and insoluble fibers (15–20%), and proteins (9–13%) [8,9]. Polyphenol contents are 1.44 g/100 g and lipids 5 g/100 g [10]. The amino acids found in pearl millet are 10.7 g/100 g of leucine, 23.0 g/100 g of glutamic acid, 8.7 g/100 g of alanine, and 5.8 g/100 g of proline [11]. The vitamin E content is 100 µg, and it has 1.8 µg of vitamin K, 842 µg of thiamine, 580 µg of riboflavin, and 768 µg of vitamin B6 [11]. Compared with other cereals such as rice, corn, and sorghum, pearl millet has nutritional potential [12] as a source of essential nutrients, including vitamins and minerals, but with higher protein and fiber contents [13].

Regarding the fatty acid profile of pearl millet grain, 77.2% are unsaturated, of which 47.5% are represented by linoleic acid. This fatty acid can contribute to cardiovascular health and, depending on the dosage, reduce LDL-cholesterol through its mechanisms of digestion and absorption, hepatic modulation, and cholesterol excretion [14]. The phenolic compounds vanillic acid, syringic acid, salicylic acid, p-OH benzoic acid, melilotic acid, o-coumaric acid, caffeic acid, protocatechuic acid, synaptic acid, and chlorogenic acid are also found in pearl millet [15]. The cereal contains 15.3 µg/g of gallic acid, 7.4 µg/g of syringic acid, 1350 µg/g of coumaric acid, and 199 µg/g of ferulic acid [16].

Pearl millet contains antinutrients, substances found in most foods that can exert beneficial effects at low concentrations [8]; however, when consumed in large quantities, these factors can compete with essential nutrients, impairing their absorption, bioavailability, and utilization. Additionally, these factors may cause stomach discomfort, nausea, and indigestion [8]. Some of the antinutritional factors found in pearl millet are phytic acid and C-glycosylflavones, such as apigenin, glucosylvitexin, glucosylo-rientin, and vitexin [17].

Although phytic acid is considered a natural antioxidant in cereals that inhibits seed oxidation, its excessive consumption may reduce the bioavailability of minerals, including zinc, calcium, and manganese [18]. Luteolin, a flavone present in pearl millet, exhibits antioxidant activity; however, other studies showed that C-glycosylflavones may inhibit thyroid peroxidase (TPO), an enzyme in the thyroid gland responsible for producing thyroid hormones [19]. Non-digestible oligosaccharides like raffinose, stachyose, and verbascose found in pearl millet may cause abdominal discomfort and flatulence [20]. Some studies suggest that pearl millet grain processing using different techniques may reduce antinutritional factors, enabling safe consumption [5,20].

Food processing involves a set of physical, chemical, and/or biological techniques applied to raw materials to transform them into safe, stable, and suitable food products for human consumption [5]. Industrial food processing is crucial to meeting the increasing global demand for secure and nutritious food. With an increasing urban population and the need to ensure food safety, the food industry plays a crucial role in large-scale production, preserving food quality for more extended periods and enabling traceability—fundamental practices that help ensure product integrity and consumer trust [21].

Pearl millet grain processing involves several operations and techniques aimed at preserving macronutrients, micronutrients, and bioactive substances, while also reducing antinutritional factors. These steps facilitate the acquisition of food with higher nutritional quality and a longer shelf life [22]. Pearl millet processing encompasses conventional and advanced techniques. Conventional pearl millet processing techniques are commonly used in the food industry, and they are divided into non-thermal—biological (fermentation, malting, and germination) and mechanical (decortication and milling)—and thermal/hydrothermal (cooking, broiling, roasting, extrusion, and parboiling) methods [23].

Furthermore, advanced pearl millet processing techniques, such as emerging thermal and non-thermal sustainable methods, are among the most advanced technologies currently employed in the food industry. These techniques have been used to extend shelf life, enhance nutritional quality, and ensure food product safety. Non-thermal techniques include cold plasma, ultrasound, high-pressure, and ultraviolet radiation [24,25,26], whereas thermal techniques include microwave and hot-air-assisted radio frequency.

One of the biggest challenges in the food industry is balancing the intensity of industrial processing with nutritional functionality, which requires developing processing strategies that promote synergy among biological, physical, and/or enzymatic steps. Most pearl millet processing studies in the scientific literature focus on improving quality attributes, including extended shelf life, sensory characteristics such as flavor and color, and nutritional quality, while maintaining or increasing the content of macro- and micronutrients and bioactive substances, and decreasing antinutritional factors. However, there is a knowledge gap in studies that extrapolate the post-processing of pearl millet to in vivo and in vitro experimental models, given that diet and nutrition are closely related to maintaining metabolic health and preventing and treating NCDs.

This study aims to demonstrate, based on current literature, how conventional and advanced pearl millet processing techniques can ensure nutritional quality and reduce antinutritional factors, evaluate how these could act on the metabolism, and finally cite the existing studies of pearl millet post-processing on metabolic health and as an adjunct in the treatment of metabolic diseases.

## 2. Materials and Methods

The study was conducted across the following databases: PubMed, ScienceDirect, Scopus, MEDLINE, SciELO, Web of Science, and LILACS. The following keywords were used in various combinations to perform the search: “pearl millet processing”, “bioactive potential and nutritional pearl millet”, “antinutritional factors of pearl millet”.

The inclusion criteria were (a) original studies that tested millet’s attributes (in vitro, in vivo and clinical), including anti-inflammatory, antihypertensive, modulation of intestinal health, antihyperglycemic, antihyperlipidemic, etc.; (b) studies conducted on how to improve the nutritional content of millet; and (c) review and original articles only in peer-reviewed periodicals, published in recent years. This narrative review did not include original articles with insufficient or incomplete data.

## 3. Results and Discussion

### 3.1. Pearl Millet Processing Techniques and the Impact on Metabolic Health

Pearl millet is frequently processed before human consumption to extend shelf life, improve nutritional quality and sensory attributes, and remove inedible portions. Pearl millet processing encompasses both conventional and advanced techniques aimed at enhancing nutritional quality, including modifications to macro- and micronutrients, bioactive compounds, and antinutritional factors.

The following section discusses the main conventional processing techniques, and the subsequent section demonstrates the effects of traditional post-processing on metabolic health in in vivo and in vitro experimental models. Finally, the last section addresses advanced processing techniques for pearl millet and how this post-processing could influence metabolic health.

### 3.2. Conventional Techniques

Conventional processing techniques, which are also known as traditional methods, are commonly used in the food industry [25]. Conventional processing techniques for pearl millet grains are categorized into biological processes (fermentation, malting, and germination) [23]; mechanical processes (decortication and milling [25]); and thermal/hydrothermal processes (cooking, roasting, and extrusion) [25].

#### 3.2.1. Non-Thermal

##### Biological

*(a)*  *Fermentation*

Fermentation is a process that obtains energy in the absence of oxygen through anaerobic activity. It is widely used in the production of flour-based products, such as breads, and supports growth through bacterial reactions. They degrade the organic matter, break the bonds between glycosidic chains, and liberate carbon dioxide (CO_2_), conferring texture, taste, and aroma to foods. Its use has been associated with improving the composition of processed pearl millet, following hygienic and sanitary requirements to prevent the release of bacterial and mold toxins [27]. The fermentation of grains acts as a prebiotic for the bacteria present in the intestine (*Lactobacillus*, *Bifidobacterium*, non-pathogenic strains of *Escherichia coli*, *Faecalibacterium prausnitzii*, *Akkermansia muciniphila*, and *Bacteroides fragilis*), supporting digestion. One study demonstrated that after fermentation and germination, reductions occur in tannins (73.4 to 26.5 mg/100 g), phytic acid (8.77 to 2.44 mg/g), and saponins (16.7 to 29.1 mg/100 g) [28]. The reduction of these compounds can be attributed to their use as carbon and energy sources by the microorganisms present in the process [27].

The reduction of phytic acid after the fermentation process can be explained through the activity of phytases, enzymes produced by fermenting microorganisms, including lactic acid bacteria and yeasts. During the fermentative process, these enzymes hydrolyze the phytic acid (or phytate), liberating inorganic phosphate ions and turning minerals such as iron, zinc, calcium, and magnesium more bioavailable. This process is favored by the acid environment produced by fermentation, which activates the phytases and enhances their efficiency. Thus, fermentation is an effective biotechnological strategy for degrading phytic acid in foods, thereby improving nutritional value [29]. Studies have highlighted this technique for increasing the levels of phenolic compounds and antioxidant potential [30,31,32,33,34,35,36].

It is essential to highlight that the association of different processes is utilized in combination to enhance the nutritional value of flours and biscuits made from pearl millet, including fermentation and malting, which improve the nutritional profiles of proteins, crude fiber, energy value, and essential and non-essential amino acids [37]. In a test with millet grains fermented to obtain flour, after drying and processing at 50 °C for 4 h, a 22% reduction in the apparent density of the flour was observed, along with improvements in attributes such as lighter color, higher peak, and final viscosity. These quality attributes indicated that the dough formed became thicker and more consistent after gelatinization [38] and could be well applied to bakery products (natural fermentation).

Another study found that fermentation time of malted pearl millet (24–72 h at 25 °C) with lactic bacteria, followed by drying at 50 °C for 10 h, influenced flour coloration. It was observed in the cultivar Agrigreen that the longer the time, the clearer the flour (L*) and the lower the a* values (red). The prolonged fermentation process may have interfered with the initial temperatures of Agrigreen flour gelatinization, the fusion peak, and the conclusion. There was also an increase in enthalpy, that is, the energy required to break the crystalline regions of the starch granules, which reduced the gelatinization range (final temperature below the start of gelatinization) [39]. In contrast, the cultivar Babala exhibited increased red and yellow tones, but enthalpy decreased with fermentation time [39].

A response surface method was used to investigate the effect of temperature (30 to 45 °C), yeast concentration (2 to 4%), and fermentation time (18 to 24 h) variables in the fermentation process of pearl millet, as well as the nutritional and phytochemical composition of pearl millet flour. The results verified that the ideal fermentation temperature was 30 °C for 18 h with 2% baking yeast (*Saccharomyces cerevisiae*). This condition resulted in the best balance among the contents of phenolic compounds, flavonoids, proteins, and sugars [35]. Another investigation on the fermentation of millet (HHB-197) with *Aspergillus awamori* MTCC-548 at 30 °C for up to 10 days found that fermentation time influenced the content of total phenolic compounds and antioxidant activity, as estimated by the DPPH and ABTS methods. A significant increase in phenolic substances occurred until the 8th day of fermentation (85.12 mg GAE/g), compared with unfermented pearl millet (18.00 mg GAE/g). In that period (8 days), the DPPH value increased from 233.36 μmol VCEAC/g (control pearl millet) to 262.7 μmol VCEAC/g, and the ABTS increased from 244.28 μmol VCEAC/g (control) to 281.86 μmol VCEAC/g [40].

Another study subjected pearl millet to fermentation at 38 °C for 0, 24, 48, 72, and 96 h in a mixture of *Lactobacillus pentosus, Lactobacillus sanfranciscensis*, and yeast strains. The results showed a higher phenol content after 72 h. Fermentation increased the content of cinnamic acids and flavonoids by approximately 30%, and the total phenols in the fermented sample were higher (206 mg/100 g), increasing after acid hydrolysis (H_2_SO_4_ 1.2 M) to 278.3 mg/100 g. This proves the importance of MP grain processing in the bioavailability of these bioactive compounds [41].

As for the applicability of processing in the functional activity of food, it is possible to mention the improvement in the digestibility of protein and starch resulting from the natural fermentation process by lactic acid bacteria in pearl millet grains. Moreover, the ability to release bioactive peptides that act on the retention of essential amino acids benefits the host’s health [42,43]. In addition, the fermentation process increases the availability of calcium, iron, phosphorus, and zinc compared to unfermented samples [44]. Furthermore, studies have shown an increase in total phenolic content in pearl millet fermented with *Rhizopus azygosporus* [45] and with a combination of yeasts and *Lactobacilli* [41].

The prebiotic action of the polysaccharide glucan, with α-D-glucopyranosyl (1→3) residues from pearl millet, has also been evaluated due to its resistance to hydrolysis by human digestive enzymes and salivary and pancreatic α-amylase. Thus, the polysaccharide derived from pearl millet may modulate intestinal homeostasis, showing promise as a functional ingredient in foods [46]. Once in the colon, the resistant polysaccharide serves as a substrate for potentially beneficial bacteria such as *Lactobacillus* and *Bifidobacterium* [47].

*(b)*  *Malting*

Malting is a technological process applied to cereal grains that involves three main steps: soaking in water (imbibition), controlled germination, and drying. During this process, grains are initially hydrated to stimulate metabolic activation, followed by a period of germination under specific temperature and moisture conditions [39]. In this phase, endogenous enzymes, such as amylases, proteases, and phytases, are activated, promoting the breakdown of complex macronutrients (starch, proteins, and phytates). This results in higher nutrient bioavailability and the accumulation of bioactive compounds, including polyphenols and antioxidants [39]. The final step of drying interrupts germination and stabilizes the product, preserving the nutritional improvements acquired [39].

Ibidapo et al. [48] demonstrated that the combination of malting, germination, and milling of pearl millet had flavonoid levels of 48.4 mg/100 g and 83.5% DPPH radical scavenging activity (DPPH). The higher phenolic content was due to the hydrolysis of condensed procyanidins. The contents of total phenolic compounds were 34.73 mg/100 g for the combined pearl millet processing flour and 19.02 mg/100 g for the wheat flour [48]. These data indicate that, though both flours contain compounds with functional potential, combined pearl millet processing flour presents a higher concentration of bioactive compounds, which demonstrates its superior technological and nutritional potential for the development of functional foods [48].

The combination of malting and fermentation processes helps reduce flour density, making it lighter and more aerated. This alteration in the grain is caused during these processes by the degradation of structural components and by the consumption of energy reserves, such as starch and sugars, by enzymes activated by malting and by microorganisms during fermentation. The longer the malting and fermentation, the greater the tendency to experience density reduction, resulting in a more porous, less compact flour that can favor the texture of final products, such as breads and cakes [39]. However, there is an optimal point to avoid sudden enzymatic alterations that could compromise the final composition. This ideal time was determined as 50.69 h for malting and 39.38 h for fermentation [39].

Sibanda et al. evaluated the combination of malting and fermented pearl millet flour, which increased the protein content by 22%, decreased the saturated fat content of pearl millet by up to 13%, increased the polyphenol content by 80%, and increased the oxygen radical absorbance capacity by 25% [49]. Another study observed that the combination of malting and germination was effective in digestion, stimulating enzymatic activity in germinated seeds, breaking down macronutrients (carbohydrates, proteins, and lipids) into simpler structures, and reducing antinutritional factors [38].

Thakur et al. [50] compared milling, roasting, and malting techniques for grain processing and the production of protein-rich beverages based on barley, pearl millet, quinoa, and yellow peas [50]. Malting increased the protein content from 11.45 to 14.9 g/100 g compared with raw grains. The combination of 30 g roasted flour of yellow peas, 28 g roasted barley pearled flour, 30 g malted pearl millet flour, and 12 g malted quinoa flour presented an energy value of 345.8 kcal [50].

*(c)*  *Germination*

Germination is a biotechnological process that involves the controlled hydration of grains, promoting the activation of cellular metabolism and the synthesis of endogenous enzymes [51]. This biotechnological process is strategically developed to enhance the nutritional and functional value of foods, leading to significant technological modifications [52]. By releasing enzymes, it promotes the partial breakdown of macronutrients, releases minerals for greater absorption, reduces antinutritional compounds, and increases levels of B vitamins and antioxidant substances [51].

Sharma et al. [51] ademonstrated that germinated pearl millet increased protein (9.16 to 10.00 g/100 g), ash (1.84 to 1.95 g/100 g), and crude fiber (1.39 to 2.11 g/100 g) contents. In contrast, there was a decrease in carbohydrate (74.99 to 72.32 g/100 g) and fat (4.12 to 3.01 g/100 g) quantity [51]. Moreover, this processing was able to increase calcium (35.76 to 71.21 mg/100 mg), magnesium (95.12 to 97.43 mg/100 mg), potassium (375.45 to 523.02 mg/100 mg), and zinc (4.04 to 4.80 mg/100 mg) and decrease iron (5.98 to 5.23 mg/100 mg), tannins (0.61 to 0.45 g/100 g), and phytates (15.11 to 6.34 mg/g) [51]. This reduction occurs due to the activation of endogenous enzymes, such as phytase and polyphenol oxidase, which degrade or transform antinutritional compounds, thereby increasing the bioavailability of minerals like iron [53]. Furthermore, germinated pearl millet showed increased DPPH percentage (55.25% to 82.42%) and total phenol (80.11 to 112.4 mg GAE/100 g) [51].

As previously cited in a study by Akinola et al. and Ibidapo et al., the combined process of pearl millet malting and germination is effective in reducing antinutritional factors and enzymatic activity, while germination, malting, and milling revealed higher flavonoid and DPPH contents, respectively [38,48]. Additionally, Theodoro et al. observed that the combined process of germination and cooking enhanced lysine, vitamin C, and iron availability in vitro while decreasing phytate, lipids, and total vitamin E when compared to a non-germinated cooked millet flour [54].

##### Mechanical

*(a)*  *Decortication*

Decortication is a step of food processing that consists of removing the external layer (or peel) from grains [5,55], seeds, or legumes. Depending on the degree of decortication of pearl millet grains, it can negatively impact nutritional value, including dietary fiber and minerals [56]. However, it can increase storage stability by reducing lipid oxidation and, consequently, preventing rancidity [57]. However, the decortication technique is insufficient to inactivate lipolytic enzymes to a significant extent, thereby impacting the nutritional attributes and antinutrients of the grains [57]. This processing also reduces the antinutritional factor, phytic acid (phytate). Phytic acid is considered a natural antioxidant in cereals, helping inhibit the oxidation of grains. High consumption of this compound may reduce the bioavailability of minerals [58] and decrease the digestibility of proteins in the digestive tract [56].

As for the type of husking, traditional manual or mechanical, few differences were recorded in terms of yield, as a moderate husking process preserves the endosperm, where nutrients are concentrated, and the bioavailability of micronutrients. However, the difficulty in determining the ideal amount of husking also presents a challenge from a nutritional point of view. This is because the lower the yield, the greater the phytate removal, which is the main chelating factor that impairs mineral absorption in the duodenum [56], making it difficult to maximize micronutrient levels.

A study compared the nutritional properties of pearl millet grains at 3% and 10% decortication levels for flour preparation. At 3%, decortication reduced antioxidant potential by 15% and total phenolic content by 25%. At 10%, it led to significant losses of zinc (17.7%) and iron (81%). There was also a 45.6% loss of phenolic compounds, reducing antioxidant potential by 38.9% and impacting the functional properties of the flour. Processing also led to 71.9% inactivation of lipase enzymes and 6.2% inactivation of lipoxygenase (LOX) [59]. It is important to note that the lipase enzyme breaks down triglycerides into free fatty acids and glycerol. At the same time, LOX catalyzes the formation of corresponding hydroperoxides from polyunsaturated fatty acids, thereby affecting the organoleptic characteristics and color of pearl millet flour [60].

Another study showed that pearl millet flour with an extraction rate of 87.5% promoted a significant reduction in the content of minerals such as iron (45%), manganese (33%), calcium (31%), zinc (22%), copper (15%), dietary fiber (41%), and protein (5%) when compared to raw flour. The phytate content found in hulled flour was 8.30 ± 0.52 (mg/g), while that of raw flour was 8.72 mg/g [61]. However, Hama et al. [56] reported that hulled millet grains with an extraction rate of 85.2% resulted in a 64% loss of phytate content in hulled flour [56].

A combination of thermal pretreatments followed by decortication has been associated with a significant reduction in phenolic compound content in millets. The heat can promote partial degradation of free phenolics and facilitate the formation of complexes with other macromolecules, such as proteins and starches, thereby reducing their extractability. In addition, decortication, a process that removes the grain’s external layers, where these compounds are more concentrated, contributes to further loss. Thus, the combined use of thermal treatments and decortication results in a considerable loss of total phenolics, negatively impacting the antioxidant potential of the grains [62].

*(b)*  *Milling*

Milling is a process in which grain is ground to produce fractions of various sizes, preparing them for the development of multiple products. It is a crucial initial step in many conventional processes to improve the efficiency of subsequent steps [5]. Grinding technology includes the traditional manual method using a pestle and mortar (a wooden pot and socket about 1.5 m long), as well as mechanical grinding. The grains are struck several times forcefully with both hands and crushed until reduced to the desired size. From this point, it can be consumed cooked, such as in soup, or roasted, such as in flour [63]. Maceration with a mortar and pestle can remove the husks from pearl millet grains and, according to a study by Hotz and Gibson, increase the bioavailability of iron, zinc, and calcium [64]. Another grinding technique involves the use of specialized stones to obtain refined cereals. In this technique, a small stone with a partially flat surface is rubbed against a large flat stone to crush grains, such as millet, transforming them into a fine flour that can be used in the preparation of many foods [65].

The duration of grinding significantly influences nutritional quality, especially iron content. A study showed that, to increase the bioavailability of this mineral, an adequate amount of β-carotene is necessary, combined with the reduction of inhibitory factors. Although sequential milling reduced these inhibitors, the bioavailability of minerals varied across hulled grains, with zinc showing the highest bioavailability [66].

Regardless of type and low cost, nutrients can be lost during milling; however, millet grains are milled with the husk, which results in a higher concentration of dietary fiber, minerals, phenolic compounds, and vitamins, yielding wholemeal flour used in the preparation of various foods. For example, a process that helps separate starch from other constituents of millet, such as starch extraction, usually involves wet-milling techniques, including water immersion and treatment in alkaline or acidic solutions [67]. Among these methods, the alkaline process is generally considered the most effective for obtaining a higher yield of millet starch, since it solubilizes the protein component and extracts the starch more efficiently [68].

However, each approach has advantages and disadvantages. Processing techniques such as grinding reduce the levels of antinutritional factors (e.g., phytic acid and tannins) concentrated in the outer layers, thereby aiding the better absorption of essential minerals, such as iron and zinc. It also improves protein digestibility. On the other hand, the milling process has disadvantages, including the loss of nutrients concentrated in the bran and germ fractions (minerals, dietary fiber, phenolic acids, and flavonoids). When bran is removed during milling, the total crude fiber content of the resulting flour decreases compared to whole grain [69,70]. In addition to reducing product stability, once millet grains are ground into flour, the seed structure disintegrates, mainly due to the high fat content (specifically unsaturated fatty acids) that is exposed during grinding, leading to rapid rancidity and the development of undesirable flavors. All of this leads to a significant reduction in service life, making it more susceptible to biological activity and environmental factors [62,71].

#### 3.2.2. Thermal and Hydrothermal

*(a)*  *Cooking*

The cooking method involves applying heat, resulting in a microbiologically safe product. This process alters the physical and chemical properties of foods, making them tastier and easier to digest [5].

In pearl millet flour obtained by roasting at 115 °C and grinding the grain, the mild thermal treatment was beneficial in preserving phenolic compounds and flavonoids and increasing antioxidant activity and ABTS sequestration (a radical to assess antioxidant activity), but it made the flour darker, with a stronger red and yellow tone. Severe thermal treatment by cooking the grain in boiling water and grinding reduced all these parameters, except metal chelation activity [72].

Heat treatment also reduces lipase activity by denaturing the enzymes. However, a test conducted with dry heat treatment in a hot air oven on pearl millet flour demonstrated that it prolonged the shelf life of the flour by 21 to 28 days. This test related storage time to lipid degradation and aimed to control fat oxidation by subjecting the grains to a temperature of 100 °C for 10 min to 2 h [73]. Another study showed a 63.6% reduction in free fatty acids in flour when millet grains were heat-treated at 100 °C for 120 min, thereby extending the shelf life of millet flour by 30 days [74].

The combination of heat treatment and laminated packaging for millet flour storage contributed to a 10- to 15-fold reduction in free fatty acids, a 2-fold decrease in peroxide value, and a 60-day increase in the product’s shelf life [75]. The combination of processes with treatments in millet flour, such as hydrothermal–hydrothermal–near-infrared radiation, compared to isolated treatments, also showed a significant decrease in the activities of lipase (47.8%), lipoxygenase (84.8%), peroxidase (98.1%), and polyphenol oxidase (100%), as well as a 67.84% and 66.4% reduction in free fatty acid and peroxide levels, respectively, in the flour, without altering the digestibility of starch and protein [76].

*(b)*  *Roasting*

Roasting is an easy process to perform that improves the edibility and digestibility of grains and their functional properties [5]. In millet flours, roasting (120 to 180 °C, 3 to 15 min) increased the bioavailability of iron, ash content, carbohydrates, and energy value but did not affect lipid, threonine, and glycine levels. Regarding phenolic compounds, the content decreases after roasting, more intensely under severe roasting [77]. Roasting is an indispensable method, as it reduces the phytic acid content, an antinutrient, by 10%, improving the bioavailability of phenolic compounds and minerals [78].

Heat treatment alters physicochemical and structural aspects, modifying the starch-protein complex and expanding the grains [79]. In addition to improving protein and carbohydrate digestibility, it can extend shelf life and reduce antinutritional elements such as phytates, tannins, and polyphenols [80]. However, reducing polyphenol levels reduces antioxidant activity [81]. Elevated temperatures can also accelerate lipid oxidation and the potential for rancidity, driven by lipolysis and fatty acid oxidation [69].

Above all, the record shows that roasting pearl millet at temperatures above 100 °C for up to 15 min increased carbohydrates and water solubility [82]. In fact, the process interfered with the functional and nutritional properties of pearl millet flour, resulting in protein contents ranging from 7.40 to 8.38%, total ash from 1.68 to 2.21%, and crude fiber from 0.50 to 1.06%. Lysine levels ranged from 0.10 to 0.26 g/100 g of protein, and methionine from 0.01 to 0.06 g/100 g of protein. Like this, samples roasted at 180 °C for 10 min showed the highest iron content, as well as increased water solubility and oil absorption capacity of the flour samples, and a significant decrease in the content of phenolic compounds [25,77].

*(c)*  *Extrusion*

Extrusion is a hydrothermal process widely used in the food industry. It includes several unit operations, such as mixing, cooking, shearing, kneading, and molding. The extrusion process inactivates enzymes and controls microbial contamination through HTST (High Temperature Short Time) [9,83]. The method is utilized as much for cooking as for structural modification of foods, resulting in products with specific characteristics of texture, form, and density [5]. During the process, the ingredients are introduced in a heated cylinder, where they are submitted to the action of one or two rotative screws [84].

This method is considered efficient and versatile, widely used in the production of breakfast cereals, snacks, infant foods, restructured meat products, functional foods, and animal feed, and it also improves palatability and digestibility [85]. The extrusion process also contributes to the microbiological food security, as high temperatures and pressures are applied during the process, inactivating pathogenic and deteriorating microorganisms such as bacteria, fungi, and yeasts. This results in more stable products with a longer shelf life and a lower risk of contamination, especially in ready-to-eat foods [85].

Among the processing methods applied to pearl millet to improve its nutritional value, thermoplastic extrusion also stands out, a process that combines high temperature (generally 60–80 °C), high pressure, and shearing forces. These conditions promote necessary physical-chemical transformations, such as the gelatinization of starch, the denaturation of proteins, and the reduction of antinutritional compounds, such as tannins and phytates. During extrusion, the mixture is forced through a matrix and, at the extruder outlet, undergoes rapid expansion due to a sudden pressure drop, resulting in a final product with an aerated, crispy texture. This type of processing not only improves the digestibility and palatability of pearl millet-based foods but also widens its applications in the formulation of functional, gluten-free products [84].

The whole flour of extruded pearl millet, cv. BRS 1502, presents higher content of phenolic compounds (131.9 ± 5.0 μmol gallic acid eq./g) and antioxidant activity (DPPH: 80.8 ± 1.6% sequestration of free radicals; FRAP: 233.7 ± 0.1 μmol Fe^2+^/g) than the raw flour (which has phenolic compound, DPPH, and FRAP values of 122.4 ± 2.05 μmol gallic acid eq./g, 69.1 ± 1.04, and 177.7 ± 1.5 μmol Fe^2+^/g, respectively) [86]. Another study identified antihyperglycemic activity in vitro (99.5 ± 0.4%) in raw and pre-cooked pearl millet flour dough using thermostatic extrusion, as well as presenting an improvement in the nutritional composition in fiber (7.0%), protein (14.8%), and lipids (8.1%), giving the product satisfactory characteristics in terms of nutritional and functional properties [87].

A previous study observed that the combination of extrusion and cooking could increase carbohydrates, essential and non-essential amino acids, iron availability in vitro, manganese, diosmin (a flavone), and cyanidin (an anthocyanin). On the other hand, extrusion-cooked pearl millet reduced total lipids, increased saturated fatty acids, decreased polyunsaturated fatty acids, and decreased dietary fiber, resistant starch, and total vitamin E [54].

**Figure 1:**
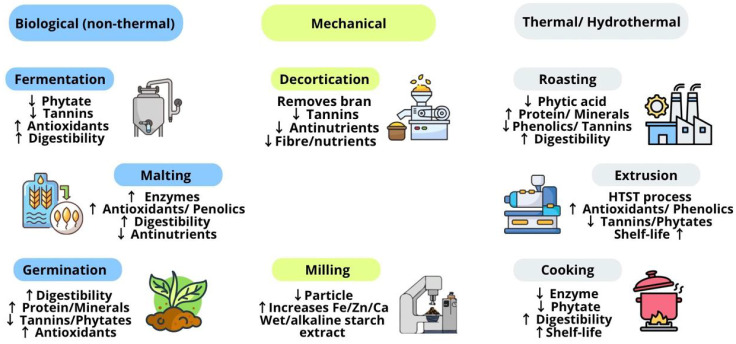
Principal conventional processing techniques of pearl millet and their influence on nutritional quality and antinutritional factors.

Figure 1 summarizes the principal conventional processing techniques of pearl millet and their influence on nutritional quality and antinutritional factors.

### 3.3. Conventional Techniques: The Impact on Metabolic Health (In Vivo and In Vitro Studies)

*(a)*  *Anti-Obesogenic and Antidiabetic Activities*

Studies performed by Theodoro et al. (2021a,b) [6] showed that germinated millet flour reduced adiposity by increasing brown adipose tissue production and lowering blood glucose levels by increasing AMPK (AMP-activated protein kinase) gene expression, inhibiting gluconeogenesis, and stimulating glucose transport. A recovery in insulin sensitivity was observed in rats fed a high-fat-fructose diet. It was also directly linked to a reduction in the inflammatory signaling pathway, decreasing the activation of NF-kB (Nuclear Factor kappa B), a transcription factor of cytokines, and TNF-α (Tumor Necrosis Factor alpha), a pro-inflammatory cytokine, as well as reducing oxidative stress [6,88]. Oxidative stress caused by free radicals contributes significantly to the development of metabolic disorders, such as chronic diseases, cardiovascular diseases, diabetes, cancer, degenerative diseases, and inflammatory diseases [89]. To neutralize free radicals and mitigate oxidative stress, stimulation of enzymatic antioxidants such as superoxide dismutase (SOD), catalase, and glutathione peroxidase [90].

As for carbohydrates, both quantity and quality are considered critical factors, as they play a key role in regulating blood sugar and preventing chronic hyperglycemia [91]. The antihyperglycemic potential of natural compounds such as phenolics (vanillic acid, syringic acid, salicylic acid, p-OH benzoic acid, melilotic acid, o-coumaric acid, caffeic acid, protocatechuic acid, synaptic acid, and chlorogenic acid) present in pearl millet grains [15] is related to the inhibition of carbohydrate digestion and the modulation of glucose uptake. The mechanism for reducing glucose levels involves inhibition of the enzymatic activities of α-glycosidases (maltase-glycoamylase and sucrose-isomaltase), which act together at the intestinal brush border to complete carbohydrate digestion. Salivary and pancreatic α-amylase, involved in starch degradation and the reduction of glucose absorption in the intestine, also play a role [92].

Carbohydrate metabolic processes—gluconeogenesis, glycogenesis, and glycogenolysis—as well as an imbalance in hepatic glucose, also increase blood glucose, leading to microvascular complications, neuropathy, nephropathy, and retinopathy [93,94]. Another relevant factor is the low/moderate glycemic index of pearl millet grains (55–69), which means they are digested slowly, thereby improving glycemic control in individuals with diabetes mellitus [95,96].

In a preclinical study conducted on hyperglycemic rats fed a supplement containing fermented pearl millet seeds for 16 days, serum glucose and cholesterol levels were reduced. It has been suggested that the mechanism underlying these results lies in the high magnesium and selenium content of the product, which regulates blood glucose levels [97] since magnesium acts as an essential cofactor for several enzymes involved in insulin secretion by pancreatic beta cells (glucokinase, ATPase, and protein kinase C) and acts in the phosphorylation of the tyrosine kinase receptor and the substrates of insulin receptors 1 and 2, phosphatidylinositol 3-kinase, and protein kinase B [98].

Magnesium also indirectly participates in reducing oxidative stress and low-grade chronic inflammation, which are involved in insulin resistance [98]. Also, adequate intake of selenium, a micronutrient essential for the activity of antioxidant enzymes (selenoproteins, such as glutathione peroxidase and selenoprotein P), plays a crucial role in the insulin signaling cascade and in maintaining lipid and glucose homeostasis [99].

*(b)*  *Anti-Inflammatory Activity*

Germinated pearl millet flour reduced metabolic inflammation in Wistar rats fed a high-fat-fructose diet by increasing interleukin-10 (IL-10) content, a cytokine with anti-inflammatory properties, while reducing the expression of TNF-α, NF-kB, and inflammatory markers, such as platelet/lymphocyte and neutrophil/lymphocyte ratios, in liver tissues [6].

Anti-inflammatory effects are associated with prebiotic properties, such as dietary fiber and resistant starch, presented in pearl millet. Germinated pearl millet flour provided 0.72 g/kg/day of dietary fiber and 0.33 g/kg/day of resistant starch [6]. Moreover, these effects are also related to polyphenols, since these compounds can regulate anti-inflammatory responses by inhibiting the NF-κB signaling pathway, which is regulated by the PI3K/Akt pathway (phosphatidylinositol 3-kinase/protein kinase B (PKB)). These crucial intracellular signaling pathways, when activated, regulate cellular processes such as proliferation, growth, cell survival, and metabolism, including transcription and protein translation. When dysregulated, they are linked to diseases such as cancer and diabetes [100,101].

Proanthocyanidins, also known as condensed tannins, contribute to the anti-inflammatory process of pearl millet grains [102]. In addition, luteolin, a flavonoid found in pearl millet grains [58], can contribute to the inhibition of the expression of inflammatory cytokines, such as interleukin-6 (IL-6) and TNF-α, and also to the reduction of nitric oxide production in lipopolysaccharide (LPS)-stimulated macrophages, highlighting the antioxidant and anti-inflammatory properties of nutricereals [103,104].

*(c)*  *Gut Health*

Regarding gut health, it is important to highlight not only its lipid quality, high dietary fiber content, and antioxidant properties, but also its gluten-free status. This characteristic, especially in pearl millet, the absence of active prolamins, plant proteins rich in proline and glutamine, facilitates digestibility and prevents autoimmune reactions in people with celiac disease, making it an excellent food choice [22].

In addition, the nutritional quality of pearl millet, due to its fiber and prebiotic properties, aids digestion and gastrointestinal motility, relieving constipation. Similarly, there is evidence that proteins and their hydrolysates may exhibit anti-ulcer activity by protecting the gastric mucosa, increasing mucus production, enhancing antioxidant enzyme activity, and reducing inflammation [105,106].

The positive impact on the gut microbiota is due to increased microbial diversity, which influences the maintenance of intestinal morphology, integrity, and function. This is achieved through prebiotic fibers and bioactive compounds, such as polyphenols, that improve nutrient absorption selectivity, as well as through decreased intestinal pH, which hinders the development and multiplication of pathogenic microorganisms [107].

Another study conducted on germinated pearl millet confirmed the prebiotic fiber’s action on the composition of the intestinal microbiota and intestinal function, reducing fecal pH and increasing the concentration of the short-chain fatty acid propionate in rats fed a high-fat, high-fructose diet [108]. The intestinal microbiota can ferment dietary fiber from pearl millet grains and produce short-chain fatty acids (SCFAs), such as butyrate, regulating the intestinal microbiota. Mainly by increasing the integrity of the muscle membrane, reducing intestinal damage, controlling inflammation, and acting to reverse early stages of fatty liver disease [109].

In view of this, we emphasize the importance of studies on technologies that aim to improve the nutritional quality of pearl millet grains and their effects on intestinal health. As clearly demonstrated in the study by Balli et al. [110], processing methods such as germination and fermentation were associated with enhanced intestinal health benefits. These benefits, resulting from the fermentation process combining the microorganisms *Saccharomyces cerevisiae* and *Campanilactobacillus paralimentarius*, included increased resistant starch (9.83 g/100 g), total phenols, minerals (calcium and iron), and growth of *Bifidobacterium breve* [110].

Another favorable combination was demonstrated by the germination, cooking, and extrusion of the grains. When subjected to three different processes—cooking in an open pan, germinating and not germinating, and cooking by extrusion—the pearl millet treatments resulted in an increase in the weight of the caecum, crypt depth, crypt thickness, and circular muscle layer width; a lower fecal pH; and a greater relative abundance of Bacteroidota. Of particular note was the group that consumed MP flour germinated and cooked in a pan, which showed lower fecal pH, better α-diversity (Chao and Shannon indices), and greater longitudinal muscle layer width [106].

Therefore, the study in rats induced to iron deficiency indicated that pearl millet germination potentially improved the composition of the intestinal microbiota and intestinal morphology [106]. Another experimental study showed that a diet containing germinated millet flour can increase gut microbiota diversity. This is due to a reduction in the genus Oscillibacter and the phylum Desulfobacterota, while the family Eggerthellaceae increased, boosting β-diversity, caecum weight, and the caecum/body weight ratio. It was observed that the intestinal histological parameters of rats fed a high-fat/high-fructose diet showed a significant increase in crypt depth and thickness and goblet cell count; a reduction in fecal pH and urinary mannitol excretion; and an increase in the concentration of SCFAs [108]. Evidence of the effect of prebiotic fiber on the intestine was also identified when arabinoxylan, a compound extracted from pearl millet bran, a by-product of the grain milling process, was used. This compound was evaluated in vitro and showed growth of Lactobacillus spp. and modulation of the intestinal microbiota, decreasing the Firmicutes concentration and increasing Bacteroidetes [111].

*(d)*  *Hepatoprotective and Antidyslipidaemic Activity*

Germinated pearl millet flour reduced triglycerides, uric acid, and alanine transferase plasma concentrations and, at the same time, reversed liver steatosis (grade 2 to grade 1) and decreased the amount of inflammatory infiltrates and collagen deposition (types I and III and total collagen) in Wistar rats fed a high-fat-fructose diet [6]. At the same time, animals that were fed with germinated pearl millet flour obtained overexpression of peroxisome proliferator-activated receptor-α (PPAR-α) in the liver, which, in turn, demonstrates an essential role in the expression of genes involved in the peroxisomal and mitochondrial β-oxidation pathways, with a critical role in the oxidation of fatty acids [6].

In addition, the phenolic compounds in pearl millet have shown hypotensive and hypocholesterolemic activities by reducing low-density lipoprotein cholesterol (LDL-c) [112]. Due to the richness in dietary fiber, unsaturated fatty acids, essential minerals, and antioxidants of pearl millet, mainly through its phytosterols (such as sterols), lipid control has been demonstrated in diabetic rats, preventing cholesterol synthesis and helping to lower LDL-c cholesterol levels [113,114].

Dietary fiber, especially the insoluble fiber found in pearl millet, helps bind bile acids in the intestine. This forces the liver to use more cholesterol from the bloodstream to produce new bile acids, interfering with the absorption of dietary fat and further contributing to a hypocholesterolemic effect [114]. The lipids in pearl millet are not only a significant source of essential fatty acids but are also known to play an important role in the transport of fat-soluble vitamins such as A, D, E, and K [115]. The high levels of polyunsaturated fatty acids, particularly linoleic acid (up to 85.51%), and monounsaturated fatty acids such as oleic acid (up to 64.47%), known to mitigate the risk of heart disease by reducing low-density lipoprotein (LDL-c) cholesterol while maintaining or increasing high-density lipoprotein (HDL-c) cholesterol, clearly position pearl millet as a healthy crop for the cardiovascular system [14,116]. It is possible to consider that the content of some phytochemicals, such as apigenin, flavonoids (myricetin, quercetin, and catechin), and lignin, may help prevent cardiovascular disease [117].

*(e)*  *Antioxidant Activity*

Specific bioactive hydrolysates derived from pearl millet demonstrated antioxidant, antilipidaemic, and antidiabetic activities by inhibiting key enzymes involved in lipid metabolism, such as pancreatic lipase and cholesterol esterase [118], breaking the ester bond of cholesterol esters, separating them into free cholesterol and fatty acids, as well as performing an essential activity for the biosynthesis of bile acids [119].

As for antioxidant action, the compounds found in pearl millet bran are feruloylated xylooligosaccharides (FXOs), which are xylooligosaccharides esterified with ferulic acid. They were obtained by enzymatic hydrolysis assisted with alkaline pre-treatment and showed potent antiglycation and antioxidant properties [120].

FXOs have antiglycation and antioxidant properties, as they sequester superoxide radicals with a ferrous ion chelation effect. To evaluate this effect, they were assessed using the DPPH assay (reagent for testing antioxidant activity, 2,2-diphenyl-1-picrylhydrazyl), ABTS assay (spectrophotometric method for measuring antioxidant capacity, 2,2′-azino-bis (3-ethylbenzothiazoline-6-sulphonic acid)), and FRAP assay (ferric reducing antioxidant power). The study indicated that FXOs at 1 mg/mL in the BSA/glucose model inhibited glycation by 54.6% in the initial phase, 62.8% in the interactive phase, and 87.7% in the final phase. Thus, FXOs can be considered a functional food due to their anti-glycation and antioxidant properties [120].

Therefore, pearl millet, through beneficial bioactive compounds such as polyphenols, flavonoids, fibers, and phytosterols, promotes benefits on metabolic health. On the other hand, antinutritional factors such as tannins and phytates can impair the bioavailability of essential minerals. Thus, it is of fundamental importance to use conventional techniques to control the effects of these antinutritional compounds and enhance the bioavailability of macronutrients and micronutrients, thereby improving the quality attributes of pearl millet grains for human consumption.

**Figure 2:**
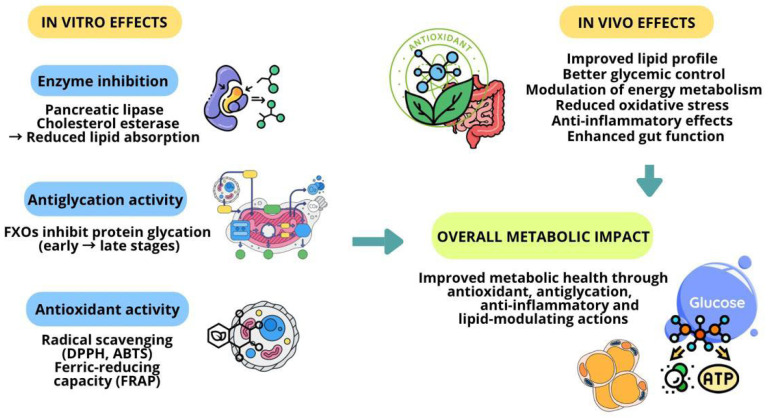
Principal studies and effects of conventional processing techniques of pearl millet and their impact on metabolic health/disorders.

Figure 2 summarizes the principal studies and effects of conventional processing techniques of pearl millet and their impact on metabolic health/disorders.

### 3.4. Advanced Techniques: Innovative Perspectives

Recent emerging technologies are considered the most innovative and sustainable techniques used in the food industry. These technologies can provide safety with minimal processing while reducing energy and water consumption, which is crucial to environmental sustainability and food security [121]. Emerging technologies are classified into two categories: non-thermal, which are the most innovative processing methods for preserving foods without substantial heating, thereby maintaining or improving the product’s nutritional and sensory attributes; and thermal, which use heat in a uniform, assisted, and controlled manner [122]. These methods are described in the following sub-sections.

#### 3.4.1. Non-Thermal

Advanced non-thermal food processing techniques ensure food safety and preservation without the need for elevated temperatures; consequently, they represent an alternative to heat-based preservation methods for maintaining food quality and safety [123]. Non-thermal processing is beneficial for preserving flavor, freshness, color, and nutritional quality, especially in thermally unstable compounds such as phenols and ascorbic acid [124]. The most common non-thermal techniques used to improve the nutritional quality of pearl millet grains include cold plasma, ultrasound, high-pressure processing, and ultraviolet irradiation [124]. These techniques are considered innovative and have attracted the attention of food manufacturers by providing a perfect balance between safety, minimal processing, economic limitations, and superior quality [125].

*(a)*  *Cold Plasma (CP)*

Cold plasma has been used in microbial inactivation and food decontamination [126]. Recently, this technique has been used to improve nutritional and antinutritional properties, rheological properties, hydration, and starch modification [127,128]. This technique uses a high-frequency plasma generator and a ceramic electrode. Plasma is a partially ionized gas composed of reactive species, including UV photons, free radicals, electrons, molecules, and excited atoms; these species can interact with proteins and alter their conformations [129].

The influence of CP on pearl millet, with input voltages of 40 and 45 kV and exposure times of 5, 10, and 15 min, on physical, nutritional, hydration, and pasting properties was evaluated. No significant differences were observed in loose and tapped bulk density; however, differences in color intensity were noted after the treatment. Moreover, crude fat content increased (3.36% to 4.71%); however, crude fiber (2.21% ± 0.02% to 1.32% ± 0.02%), protein (10.064% ± 0.43% to 8.89% ± 0.50%), and moisture decreased (11.92% ± 0.10% to 10.25% ± 0.03%) [127].

Another study investigated the effects of CP at different power levels (20–30 kV) for 10–20 min on the nutritional, antinutritional, functional, thermal, rheological, and structural properties of pearl millet flour [128]. A decrease in antinutritional factors, such as tannins and phytic acid, was observed, along with significant changes in the flour starch granular structure, resulting in a reduction in relative crystallinity from 23.45% to 19.31%. Pearl millet flour showed shear-thinning behavior in high-intensity CP-treated samples and exhibited an elastic response with higher storage modulus values (G’). Moreover, an increase in water absorption capacity (1.32 to 1.62 g/g), oil absorption capacity (1.11 to 1.31 g/g), emulsifying capacity (EC; 86.58% to 91.25%), and foaming capacity (10.67% to 12.84%), as well as a decrease in pH (7.83 to 6.97), were observed [128].

Charu et al. demonstrated that CP at 2 kV for 5 min decreased tannin and phytic acid by 18% and 57%, respectively, and total flavonoid (303.21 ± 0.21 to 271.1 ± 1.08) and phenolic content (84.03 ± 0.075 to 82.28 ± 0.03). However, this treatment enhanced fat content (2780 ± 0.380 to 3.983 ± 0.258) [130]. Altogether, the CP technique cited in the current studies could improve quality attributes, including reduced antinutritional factors, increased crude fat content, oil and water absorption, and enhanced foaming and emulsifying capacity. On the other hand, CP decreased the protein and crude fiber content, which are essential nutrients for a healthy human body.

In situations where antinutritional factors are present at high concentrations in the body, malabsorption and a consequent deficiency in the intake of minerals, vitamins, and proteins can occur, leading to a negative response from the body. Although minerals and vitamins are found in small quantities in the body, adequate micronutrient levels are critical for normal metabolic function [131].

Micronutrients such as complex B vitamins, vitamin C, and some minerals (iron and magnesium) are involved in some steps of the energy-yielding metabolism system within the cell, and vitamins B1, B2, B3, B5, and C act as cofactors [132]. Macronutrients are oxidized into acetyl-CoA in several pathways, such as glycolysis, which produces pyruvate from glucose; therefore, acetyl-CoA enters the citric acid cycle, producing energy as nicotinamide adenine dinucleotide (NADH) and flavin adenine dinucleotide (reduced) (FADH2) by eight oxidations that require vitamins B1, B2, B3, B5, B6, B8, B12 and iron and magnesium. After this process, electrons from NADH and FADH2 are transferred to the electron transport chain, which uses this energy to produce ATP. This final process requires the intake of vitamins B2, B3, and B5, as well as iron [132].

Moreover, vitamins B12 and C, as well as other essential minerals such as magnesium and zinc, help mitigate oxidative stress. These vitamins and minerals play a crucial role in developing a powerful antioxidant defense system. For example, vitamin B12 plays a protective role against lipid oxidation, primarily by participating in the glutathione (GSH) redox cycle, an enzyme with antioxidant potential that acts as an electron donor [132]. Vitamin C acts as an electron donor for other molecules; therefore, it scavenges reactive oxygen species [132]. In addition, magnesium also belongs to the antioxidant defense system, and the lack of it can result in oxidative stress since inflammation associated with low levels of magnesium can enhance the production of free radicals in phagocytes and neutrophils, leading to endothelial dysfunction [133,134].

Zinc possesses antioxidant properties that can inhibit the oxidation of macromolecules, including nucleic acids and proteins, by interacting with many enzymes critical to the antioxidant defense system, such as metallothionein, and by increasing catalase activity [135]. This enzyme scavenges reactive oxygen species. Zinc is a cofactor of superoxide dismutase, an enzyme that neutralizes superoxide anions, producing hydrogen peroxide, which is subsequently metabolized by catalase. Additionally, zinc modulates nuclear factor-κB, a transcription factor that regulates the expression of several genes involved in immune and inflammatory responses [135]. All these findings reinforce the importance of micronutrients; therefore, reducing the high levels of antinutritional factors in CP techniques for pearl millet grains is essential to provide optimal amounts of vitamins and minerals, which play a crucial role in maintaining metabolic health.

*(b)*  *Ultrasound or Ultrasonication (US)*

Ultrasound is a sound wave with a high frequency that exceeds the range of human audible frequency (20 kHz) and is commonly used in combination with temperature (thermosonication), pressure (manosonication), or both (manothermosonication) to enhance effectiveness and diminish processing time [136]. The US is classified in the food industry into two categories: low-intensity (<1 W cm^−2^) or high-frequency (>100 kHz), and high-intensity (10–100 W cm^−2^) or low-frequency (20–100 kHz) [136]. A recent study evaluated the effect of US-assisted at 44 Hz for 20 min at 30 °C on pearl millet starch-germ complexing. 30% of this complex was also incorporated in bread, and, therefore, a decrease in the glycemic index and an increase in hardness were observed in the product. The US processing of starch-germ generated microchannel formation, and the starch interacted with protein and lipids, resulting in ternary complexes. The results found higher values of the complexing index (CI) ratio (1045/1020 cm^−1^), relative crystallinity, and resistant starch (RS), which are essential parameters for the starch quality [137].

Moreover, the same group evaluated the effects of the US in combination with dry heat in pearl millet flour. They demonstrated that the combination of two technologies was able to increase resistant starch content (from 14.49% to 31.69%) and simultaneously decrease the glycemic index (from 58.25% to 48.49%) [138]. RS can act as a fiber and is more resistant to hydrolysis in the small intestine; therefore, it is a type of starch used to reduce the glycemic index in food formulations without interfering with product quality attributes [139]. RS can alter the post-meal glycemic response while preserving pancreatic beta-cell function, regulating the insulin response to maintain homeostasis in healthy individuals and in obesity and type 2 diabetes [139].

Another study investigated the effects of US treatment followed by fermentation on functional properties and bioactive compounds in pearl millet grains [140]. Different temperatures (20, 50, and 60 °C) were used in the US treatment of pearl millet grains; therefore, it was observed that the US alone and followed by fermentation were able to improve phenolic and flavonoid contents as well as antioxidant activities; similar findings were found in total carotenoids when used alone or followed by fermentation, being the highest value (78.5 µg/g) of the total carotenoid in the US-treated grains followed by fermentation at 60 °C. US treatment at 60 °C enhanced protein solubility (41.9%) of the grains. The protein solubility after US treatment alone, followed by fermentation, was improved. Altogether, the principal component analysis shows the interrelationships between US treatment at different temperatures, or US followed by fermentation; therefore, it was observed that combined US treatment and fermentation could increase antioxidant activity, improve functional characteristics, such as water-holding, oil-holding capacities, and emulsion and foaming activity/stability, and enhance phenolic, flavonoid, and carotenoid contents [140].

The ability to enhance phenolic compounds is crucial for maintaining metabolic health, as these substances are secondary metabolites of plant metabolism and act as antioxidants, intercepting free radicals before they cause cellular damage [141]. Moreover, they can inhibit lipid peroxidation associated with numerous pathological processes, including cardiovascular diseases and neurodegenerative disorders, thereby protecting the body against DNA damage, reducing inflammation, and exhibiting anticancer potential [141].

*(c)*  *High-Pressure (HPP)*

High-pressure processing (HPP) is a non-thermal method that uses pressure to inactivate microorganisms and enzymes. This technique preserves nutritional and sensory characteristics [23]. HPP causes stress on the cells, partially degrading their structure. Thus, HPP activates certain enzymes that decrease antinutritional factors [142]. The food is exposed for a certain amount of time under pressure ranging from 200 to 600 MPa [143] or 100 to 1000 MPa [144].

Himashree and Mahendran [142] evaluated the effects of HPP, combined with soaking in pearl millet, at three pressure levels (300, 350, and 400 MPa) and three soaking times (30, 60, and 90 min) to decrease phytate and simultaneously increase iron bioavailability. It was observed that the treatment reduced the phytate content by 80.61% at 400 MPa after 90 min, while simultaneously increasing the iron content from 127.73 mg kg^−1^ to 579.24 mg kg^−1^ under 350 MPa for a 90 min soaking period. No differences were observed in the nutritional profile (protein, fat, carbohydrates, and ash), while the treated samples showed increased water absorption (12.1%) and oil absorption (5.9%) [142]. Increasing iron intake is vital, as iron is an essential mineral whose deficiency can cause anemia, as it is a cofactor in oxygen transport and energy-yielding metabolism. This mineral is found in hemoglobin, a heme-containing protein in red blood cells. This mineral is part of the porphyrin ring structure of heme enzymes, such as those in the cytochrome family, which are essential for energy production [132].

Another study evaluated the effect of US- and HPP-assisted extraction of pearl millet protein isolate, achieving a recovery rate of 63.43% and improving its functional properties, including emulsion properties, solubility, and water and oil absorption capacity. Moreover, the treatment altered the protein’s secondary structure, decreasing α-helix content, increasing β-sheet content, and improving protein in vitro digestibility [145]. This study highlights the importance of using emerging technologies to improve the quality of protein from pearl millet.

*(d)*  *Ultraviolet (UV) Irradiation*

Ultraviolet (UV) irradiation has been previously used to reduce microbial contamination, thereby enhancing food safety and extending shelf life [146]. Recently, the UV-C technique has emerged as a novel food processing method that operates without heat [70]. Specific molecules in pearl millet grain can be targeted by UV-C irradiation, thereby diminishing antinutritional factors such as tannins and phytates and maintaining nutritional value [70]. UV-C irradiation reduced total protein content with increasing treatment intensity. A decrease in total phenolic compounds was observed in prolonged exposure. Even at reduced UV intensities, phytates decrease with increasing exposure duration and bed density. A reduced trend in lipase activity was observed with increases in UV power and exposure duration, as well as with reduced bed density [146].

Lipase is a lipolytic enzyme that hydrolyzes glycerides into free fatty acids (FFAs), leading to rancidity, bitterness, and off-flavors [75]. When UV power and exposure duration were extended, the whiteness value of the flour enhanced [146]. Although no studies were found that used UV-C-irradiated pearl millet processing in in vivo and in vitro models to investigate metabolic health and disease, this study suggests that the UV-C irradiation method improves specific quality attributes, including reduced phytate levels and lipase activity, and increases flour whiteness. However, the nutritional quality, including protein and phenolic compound content, decreased as the intensity and duration of the treatment increased.

As described in previous studies, reducing antinutritional factors at high concentrations can improve the bioavailability of micronutrients, such as minerals and vitamins, which are essential to metabolic health and associated disorders, especially complex B vitamins, vitamin C, iron, magnesium, and zinc, that are involved in energy-yielding metabolism and a powerful antioxidant defense system [132].

#### 3.4.2. Thermal

Advanced thermal food processing techniques are employed to enhance the quality attributes of pearl millet products, thereby imparting physical, sensory, and functional properties suitable for various food applications. The most common advanced thermal methods reported in the current literature for pearl millet include hot-air-assisted radio frequency (HARF) [147] and microwave [148] techniques.

*(a)*  *Hot-Air-Assisted Radio Frequency (HARF)*

Radio frequency (RF) is a non-ionizing electromagnetic wave that produces heat from molecular friction between water molecules in the interior of the material due to ionic conductivity and/or dipole rotation. Compared with microwaves, RF waves have longer wavelengths and greater depth penetration, which facilitates the treatment of bulk food materials [149]. HARF is an assisted technique that uses radiofrequency energy to achieve uniform heating and enhance treatment efficiency [150].

Yarrakula et al. [147] demonstrated that pearl millet at different moisture levels (10.5%, 12%, and 15%) was exposed to HARF treatment for 5, 10, and 15 min at a fixed electrode position. The findings revealed that pearl millet flour treated with HARF showed lower peroxide and free fatty acid values than decorticated raw flour (the untreated group); consequently, the storage stability of pearl millet flour was enhanced for up to 180 days. No differences were observed in the emulsifying, foaming, and cooking properties of pearl millet flour treated with HARF. The chemical composition revealed that HARF-treated pearl millets with higher moisture (15%) and exposure time (15 min), when compared to lower moisture (10.5%) and exposure time (10 min), had increased protein (5.12 to 7.11%) and ash (0.01% to 0.02%) contents. In comparison, the fat content decreased (3.83% to 2.22%), which could explain the reduced likelihood of rancidity development, and carbohydrates also decreased (91.03% to 90.63%) [147]. The mineral profile was not evaluated in the Yarrakula et al. study [147]; however, ash content increased after HARF treatment. Minerals are essential elements in metabolic homeostasis, with zinc, magnesium, selenium, and iron being important examples in energy metabolism and antioxidant activity, as demonstrated by Tardy et al. [132].

Although the amino acid profile was not evaluated in the current study, the protein content in pearl millet increased after HARF treatment. Proteins are chains of amino acids and are connected by peptide bonds. Amino acids can be classified as essential, meaning those that are not synthesized in the human body and are obtained through the diet (i.e., isoleucine, leucine, lysine, methionine, phenylalanine, threonine, valine, histidine, and tryptophan) [131]. Food that provides essential amino acids is necessary to increase muscle and whole-body protein synthesis. Skeletal muscle accounts for a considerable amount of protein in the body; therefore, protein intake and resistance exercise are essential strategies for promoting skeletal muscle hypertrophy and remodeling [151].

Proteins exhibit structural activity, and a crucial aspect is that when intrinsic mechanisms and physical activity enhance skeletal muscle, it confers a significant advantage that benefits glucose homeostasis [151,152]. Consequently, when there is an increase in muscle growth, there will be a beneficial effect on fat metabolism, since there will be an increase in adiponectin, a protein with anti-inflammatory properties and an insulin sensitizer, which is currently accepted as a myokine, a type of protein with a critical metabolic role synthesized in the skeletal muscle [152].

*(b)*  *Microwave (MW)*

Microwave, a treatment that utilizes gamma irradiation, is a type of heating treatment that offers precise control over the heat. It is used to decrease the activities of lipase and lipoxygenase enzymes, which are responsible for the rancidity process. MW treatment uses heat by rapidly agitating food polar molecules when exposed to an electromagnetic field [148]. MW treatment improved some quality attributes of pearl millet flour. It was used at three power levels (100, 200, and 300 watts) over three periods (30, 60, and 90 s). Therefore, it was observed that lipase activity (219.76 to 31.98 µg oleic acid mL^−1^), phytates (6.815 to 5.704 mg·g^−1^) and tannins (1.12 to 0.142 mg CE·g^−1^) decreased after higher power levels and exposure time. Moreover, antioxidant activity (94.06 to 93.06%), total flavonoid content (2.75 to 1.23 mg·g^−1^), and protein (10.34 ± 0.28 to 8.38 ± 0.07 g·100 g^−1^) were reduced [148]. Reducing antinutritional factors is crucial for enhancing the bioavailability of micronutrients, such as minerals and vitamins, which are essential for maintaining metabolic health [132].

**Figure 3:**
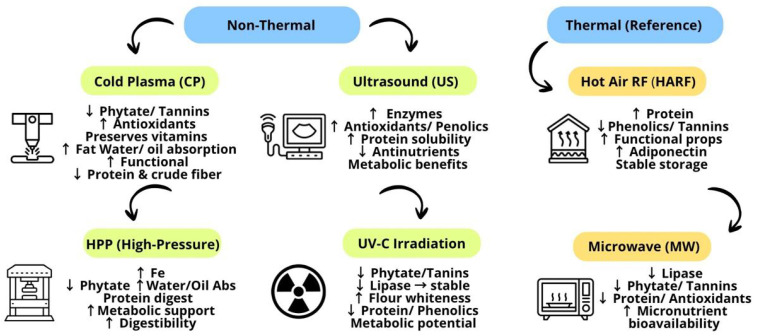
Principal advanced processing techniques of pearl millet on nutritional quality and antinutritional factors.

Figure 3 summarizes the principal advanced processing techniques of pearl millet on nutritional quality and antinutritional factors.

## 4. Conclusions

Pearl millet is a nutricereal potentially indicated for regular human consumption due to its crucial nutritional and functional properties, as well as its benefits in controlling chronic non-communicable diseases (NCDs), intestinal diseases, micronutrient deficiencies, and protein malnutrition. To achieve this, both conventional and advanced pearl millet processing are necessary to mitigate the effects of antinutritional factors and increase the bioavailability and bioaccessibility of macro- and micronutrients. It is essential to highlight that most studies evaluating the post-processing product of pearl millet in in vivo and in vitro studies and its impact on metabolic health have used conventional processing techniques, with prominent results in isolated germination, germination in combination with cooking, isolated extrusion, and extrusion with cooking.

However, some limitations still exist in this study, as no studies have yet been found that utilize advanced processing techniques for pearl millet, and extrapolation is made to studies with metabolic disorders or health in metabolism. In this way, it is necessary to apply this knowledge in preclinical dietary intervention trials in disease models to establish safety and an appropriate adjuvant therapeutic indication.

## Data Availability

No new data were created or analyzed in this study. Data sharing is not applicable to this article.
